# Secular Trends in Admissions and Mortality Rates from Diabetes Mellitus in the Central Belt of Ghana: A 31-Year Review

**DOI:** 10.1371/journal.pone.0165905

**Published:** 2016-11-22

**Authors:** Osei Sarfo-Kantanka, Fred Stephen Sarfo, Eunice Oparebea Ansah, Benjamin Eghan, Nana Kwame Ayisi-Boateng, Emmanuel Acheamfour-Akowuah

**Affiliations:** 1 Directorate of Medicine, Komfo Anokye Teaching Hospital, Kumasi, Ghana; 2 Department of Medicine, School of Medical Sciences, Kwame Nkrumah University of Science and Technology, Kumasi, Ghana; 3 University Health Services, Kwame Nkrumah University of Science and Technology, Kumasi, Ghana; Yokohama City University, JAPAN

## Abstract

**Introduction:**

Diabetes Mellitus is currently a leading cause of morbidity and mortality throughout the world, particularly in sub-Saharan Africa where a significant proportion of diabetes cases are now found. Longitudinal profiling of in-patient admissions and mortality trends from diabetes provide useful insights into the magnitude of the burden of diabetes, serve as a sentinel on the state of out-patient diabetes care and provide effective tools for planning, delivering and evaluating the health care needs relating to the disease in sub-Saharan Africa.

**Objective:**

To evaluate the 31-year trend in diabetic admissions and mortality rates in central Ghana.

**Methods:**

This is a retrospective analysis of data on diabetes admissions and deaths at a tertiary referral hospital in central Ghana between 1983 and 2014. Rates of diabetes admissions or deaths were expressed as diabetes admissions or deaths divided by the total number of admissions or deaths respectively. Yearly crude fatality rates for diabetes were calculated. Trends were analysed for in patient diabetes admissions and mortality for the period. Predictors of diabetes mortality were determined using multiple logistic regression.

**Results:**

A total of 11,414 diabetes patients were admitted over the period with a female predominance; female:male ratio of 1.3:1.0. Over the study period, diabetes admission rates increased significantly from 2.36 per 1000 admissions in 1983 to 14.94 per 1000 admissions in 2014 (p<0.0001for linear trend), representing a 633% rise over the 31-year period. In-patient diabetes fatality rates increased from 7.6 per 1000 deaths in 1983 to 30 per 1000 deaths in 2012. The average 28-day mortality rate was 18.5%.

The median age of patients increased significantly over the period. So was the proportion of females admitted over the years. Predictors of in-patient mortality were increasing age- aOR of 1.23 (CI: 1.15–1.32) for age > 80 years compared with < 20 years, admissions in 2000s compared to 1980s-aOR of 1.56 (1.21–2.01), male gender-aOR of 1.45 (1.19–1.61), the presence of glycemic complications such as ketoacidosis- aOR-2.67(CI: 2.21–3.21), hyperosmolar hyperglycemic states- aOR 1.52 (1.33–1.73) symptomatic hypoglycemia- aOR 1.64 (1.24–2.17) and presence of end organ complications including peripheral neuropathic ulcers- aOR 1.31 (1.12–1.56), nephropathy- aOR -1.11 (1.03–1.23), cerebrovascular disease—aOR-1.52 (1.32–1.98), coronary artery disease- aOR-3.21 (1.91–5.15) and peripheral artery disease- aOR-1.15 (1.12–1.21) were associated with increased risk of death compared with normoglycemic diabetic admissions and admissions without end organ complications respectively.

**Conclusion:**

Diabetes admission and mortality rates have increased significantly over the past three decades in central Ghana. More intensive education on the risk factors for diabetes, acute diabetes care as well as instituting hospital guidelines for diabetes control and reduction of modifiable risk factors for diabetes are urgently needed to reduce the poor case fatality associated with diabetes in resource-limited settings.

## Introduction

Most recent estimates show increasing global trends in the prevalence of diabetes and other non-communicable diseases [1, 2]. More than 80% of the worldwide increase in diabetes is expected to occur in low and middle-income countries, with the greatest relative increase predicted to occur in the urban populations of sub-Saharan Africa [3–5]. Increasing urbanisation and westernisation of populations of sub-Saharan Africa as well as a significant genetic predisposition underlies the increasing number of people with diabetes in sub-Saharan Africa [6–9] With most of the limited resources of sub-Saharan African countries dedicated towards combating infections like HIV, malaria and tuberculosis, little attention is paid to non-communicable diseases such as diabetes and hypertension in terms of allocation of funds for treatment and personnel training [10,11]. For instance, the expenditure on diabetes in sub-Saharan Africa constitutes less than 1% of the global health expenditure compared with > 48% for high-income countries in North America [12].

The lack of attention to the burgeoning epidemic of non-communicable diseases in low and middle-income countries has resulted in significant gaps in the management of these disorders often culminating in preventable deaths and disabilities. Indeed, community-based studies in the 1960’s in Ghana reported diabetes prevalence of 0.2% compared with 6.4% in 2000’s [8,9]. Furthermore, hospital-based studies have indicated a two-fold increase in diabetes in-patient admissions in the 1990’s compared to the 1980’s [13,14]. According to World Health Organisation reports, diabetes represents the sixth commonest medical cause of death in Ghana, representing 2.58% of total deaths with an age-adjusted death rate of 36.81 per 100,000 of the population [15].

Hospital-based data although not entirely representative of the community-based setting, is helpful in providing evidence base for in-patient mortality as well as reflecting the level of out-patient care for diabetes patients [16]. Understanding the temporal changes in diabetes admissions and mortality as well as demographic predictors of diabetes outcomes would be instrumental in advising policy makers in allocation of limited resources for the treatment of diabetes as well as formulating treatment guidelines and shaping the future directions of diabetes management in resource-limited settings. The aim of this study was to assess the temporal trends in demography and mortality of diabetes admissions from records at a tertiary referral hospital in Kumasi, situated in the middle belt of Ghana between 1983 and 2014.

## Methods

This was a retrospective study conducted at the Komfo Anokye Teaching Hospital (KATH), in Kumasi, Ghana. Komfo Anokye Teaching Hospital is a 1000 bed hospital located at the middle part of Ghana. It is a leading tertiary referral hospital serving an estimated population of 10 million from 6 out of 10 administration regions of Ghana as well as other neighboring countries. A review of hospital admission and deaths from 1983 to 2014 was performed at the hospital registry. Diabetes admissions within the period were obtained from tally cards and the relevant information extracted unto a questionnaire. Diabetes type and complication were recorded and classified using the International Classification of Diseases (ICD) codes ICD-9 (from 1983–1996) and ICD -10 (from 1997 to 2014). Among data recorded on diabetes tally cards were age, gender, date of admission, date of discharge or death, complications and these were entered into excel sheets by data entry clerks. Time to discharge or death was calculated by subtracting the date of admission from the date of discharge or death.

### Ethical Approval and consent

The study was approved by the Committee on Human Research Publication and Ethics of the School of Medical Sciences, Kwame Nkrumah University of Science and Technology, and the Komfo Anokye Teaching Hospital, Kumasi. Patient records/information were anonymized and de-identified prior to analysis.

### Statistical analysis

Means and medians were compared using either the Student t-test or Mann Whitney U-test for paired comparisons and ANOVA or Kruskal Wallis test for more than two group comparisons depending on whether continuous variables were parametric or non-parametric. Rates of diabetes admissions or mortality were expressed as diabetes admissions or deaths divided by total number of hospital admissions or deaths respectively. Yearly crude fatality rates from diabetes was calculated by dividing the number of diabetes related deaths by the number of diabetes admissions. Patients with >28 days’ stay were censored. A Poisson regression model was used to examine the temporal trends in the rates of diabetes with categorical year variables. Exact Wilcoxon test for ordered contingency tables, in the case of categorical variables were used to study time trends. Predictors of in-patient diabetes mortality were assessed using a multivariate logistic regression model. Variables included in the model were: age at admission in years, gender, decade of admission (1983–1989, 1990–1999, 2000–2009, and 2010–2014) and type of glycemic complications. In bivariate analyses, a p-value of 0.10 was set for selection of variables into the final multivariable model with visual inspection for compliance with collinearity assumption. A two-sided p-value of <0.05 was considered significant in all statistical analysis with no adjustments made for multiple comparisons.

## Results

### Diabetes admission rates and demography

**Fig 1:**
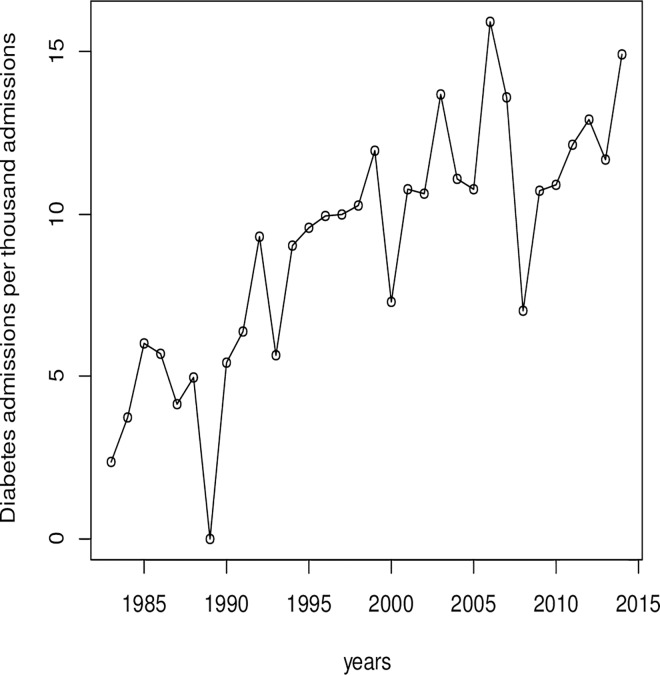
Diabetes admissions per thousand hospital admissions in Central Ghana, 1983 to 2014.

Fig 1. shows the trend of diabetes admissions per one thousand hospital admissions from 1983 through to 2014. Generally, there was an increasing trend in admissions for diabetes per every one thousand (1000) admissions in the central belt of Ghana. However, there was a progressive increase in the admissions of diabetes per thousand admissions from 1983 to 1985 which then dropped slightly from 1985 to 1987 and after increased slightly in 1988. In 2005, the admissions increased to more than 15 per thousand admissions but decreased sharply after 2005 to around 6 in 2007. However, the admissions increased steadily from 2008 through to 2014. The number of diabetes admissions per 1000 hospital admissions as shown in Table 1 indicated an overall increase in the proportion of diabetes related hospital admissions. The rate of diabetes admissions in 2014 of 14.95/1000 admissions compared to 2.36/1000 admissions in 1983 represents an increase of 633% over the period. The mean age of diabetes patients admitted also increased from 46.1 ± 16.5 years in 1983 to 59.8 ± 30.0 years in 2013. From 1983–1993, males formed a higher proportion of diabetic patients admitted, from 1994 to 2014 however, a higher number of females were admitted compared to males. The total number of female patients admitted over the period was 6399 with number of males being 4798, a female: male ratio of 1.3:1. Females patients admitted were relatively older than their male counterparts with significant difference in age emerging in 1983, 2000 and 2003.

**Table 1:** Demography and Admission Rates of Diabetes at Komfo Anokye Teaching Hospital from 1983 to 2014.

Year	No. of admissions for the year	No. of diabetes admissions	Diabetes admissions per 1,000 admissions	% Female diabetes	Mean ± SD age of diabetes patients	Mean ± SD age of males	Mean ± SD age of females	p-value
				Patients				
1983	28,772	68	2.36	47.1	46.1 ± 16.5	48.6 ± 20.2	43.1 ± 3.6	0.16
1984	31,237	116	3.71	44.8	44.4 ± 17.1	45.7 ± 19.5	42.7 ± 20.8	0.35
1985	33,969	204	6.01	43.6	46.0± 17.5	47.5 ± 16.0	44.1 ± 18.9	0.16
1986	37,387	211	5.7	45.8	52.2 ± 13.9	54.2 ± 9.4	47.5 ± 19.6	0.02
1987	37,234	155	4.16	48.4	42.8 ± 19.4	45.1 ± 20.3	40.4 ± 13.6	0.13
1988	37,049	184	4.97	44.5	46.6 ± 19.4	47.2 ± 19.9	45.9 ± 20.1	0.62
1989	38,057		-	-	-			
1990	37,584	204	5.43	45.6	47.0 ± 18.6	47.9 ± 18.5	46.1 ± 18.4	0.49
1991	36,813	234	6.36	49.9	45.3 ± 18.3	45.9 ± 15.5	45.6 ± 18.7	0.31
1992	34,317	262	9.32	42.4	48.7 ± 17.7	47.0 ± 16.0	51.3 ± 15.2	0.05
1993	37,861	213	5.63	46.1	49.2 ± 17.6	48.9 ± 14.6	49.5 ± 16.3	0.77
1994	35,702	323	9.05	56.1	60.6 ± 15.9	59.6 ± 15.7	61.5 ± 16.0	0.27
1995	35,232	337	9.57	57.3	54.0 ± 16.6	55.9 ± 15.8	52.6 ± 17.0	0.14
1996	34,855	365	9.93	52.5	51.0 ± 16.8	50.9 ± 16.9	51.1 ± 16.8	0.93
1997	39,732	318	9.99	50.9	51.3 ± 16.6	50.6 ± 17.1	52.0 ± 15.9	0.37
1998	36,987	380	10.27	51.4	50.7 ± 20.4	51.0 ± 15.2	50.4 ± 18.0	0.77
1999	34,750	417	11.97	70.4	52.7± 19.8	53.2 ± 15.3	52.4± 12.5	0.65
2000	40,601	296	7.29	50.7	51.5 ± 18.7	49.1 ±15.6	54.1 ± 11.2	0.02
2001	41,206	444	10.78	55.9	53.1 ± 19.8	53.2 ± 14.2	52.9 ± 12.5	0.86
2002	42,608	545	10.63	55.6	55.7 ± 17.3	55.9 ± 17.4	55.5± 17.3	0.73
2003	42,375	578	13.69	55.9	57.91 ± 17.0	56.4± 17.4	59.7 ± 15.9	0.02
2004	42,359	484	11.09	57.6	58.3 ± 16.2	56.5 ± 17.3	59.7 ± 15.2	0.03
2005	41,992	452	10.76	56.2	54.2 ± 17.7	54.0 ± 15.0	54.6 ± 17.7	0.58
2006	37,134	591	15.92	53.5	53.7 ± 15.2	51.9 ± 15.0	54.8 ± 15.4	0.06
2007	44,046	405	13.58	57.3	57.7± 16.6	56.6.± 15.2	58.4 ± 17.5	0.07
2008	43,934	308	7.01	59.7	59.7 ± 15.8	58.7 ± 14.3	60.5 ± 16.6	0.32
2009	41,657	446	10.7	53.3	59.0 ±17.7	59.1 ± 18.4	59.0± 17.2	0.93
2010	44,972	612	10.9	59.8	58.1 ± 17.8	57.2 ± 11.5	58.7.7±16.6	0.31
2011	42,480	516	12.15	61.5	58.0 ±17.3	58.2 ±18.1	57.8± 20.5	0.49
2012	39,436	571	12.93	55.1	58.7±16.5	59.2± 14.6	58.1± 17.7	0.41
2013	38,568	593	11.69	63.7	59.8 ± 30.0	61.0 ±16.3	59.0±15.8	0.44
2014	38,281	579	14.94	56.9	59.2 ± 16.3	58.7 ± 14.1	59.61± 16.9	0.52

### Trends in demography and complications associated with diabetes admissions

#### Demography

As shown in Table 2, there was a significant progressive increase over the decades in diabetes admissions both in absolute numbers and in the increase in the rate per overall admissions (p<0.0001). The proportion of female diabetes admissions also increased over the decades significantly (p<0.0001).

**Table 2:** Trends in Demographic and Clinical Characteristics of Diabetes Admissions in Central Ghana from 1983–2014.

Characteristic		1983–1988	1990–1999	2000–2009	2010–2014	P for linear trend
Number		938	3053	4549	2871	< 0.0001
Diabetes admissions	Per 1000 overall admissions	4.49	6.53	11.15	12.52	< 0.0001
Age (years)	Mean (SD)	48.9(17.7)	50.1(18.4)	55.7(18.4)	58.5(16.9)	<0.0001
	Median (IQR)	50(34–60)	52(38–64)	58 (45–70)	60(48–72)	<0.0001
Females N (%)		430(45.8)	1593(52.2)	2781(61.1)	1826(63.6)	< 0.0001
Type of Complication	Glycaemic	770(82.1)	2513(82.3)	3391(77.0)	2348(81.8)	
	End organ complication	168(17.9)	540(17.7)	1046(23.0)	638(18.2)	0.13
Type of Glycaemic complication	Diabetes Ketoacidosis	93(12.1)	262(10.4)	260(7.4)	351(14.9)	0.21
	Hyperosmolar State	85(11.0)	633(25.2)	1049(29.9)	587(25.0)	0.13
	Hyperglycemia	488(63.4)	1050(44.9)	1601(46.7)	1030(43.9)	0.09
	Hypoglycemia	104(13.5)	490(19.5)	559(16.0)	380(16.2)	0.12
Type of end organ complication	Peripheral neuropathy/ Foot ulcers	55(32.7)	50.1(18.4)	102(9.8)	118(18.5)	0.16
	Nephropathy	46(27.4)	358(66.3)	481(46.0)	322(50.5)	0.26
	Cerebrovascular diseases	18(10.7)	59(10.9)	109(10.4)	96(15.0)	0.11
	Coronary artery diseases	9(5.4)	14(2.6)	102(9.8)	33(5.2)	0.22
	Peripheral vascular diseases	40(23.8)	109(20.2)	252(24.1)	102(16.0)	0.16
Diabetes Case fatality rate		15.12	18.09	21.55	16.38	0.12
28-day mortality		142 (15.1)	542(17.8)	549(20.9)	458(15.2%)	0.11
Duration of hospitalisation		9(5–15)	8(4–14)	6(3–9)	4(2–10)	<0.0001

#### Glycemic complications

Of the total number of diabetes admissions, 8020 (79.0%) presented with glycemic complications. Out of this number, 2354 (26.1%) presented with hyperglycemic hyperosmolar state (HHS), 966 (10.7%) with diabetes ketoacidosis (DKA), 4,167 (46.2%) with hyperglycemia and 1533 (17.0%) with hypoglycemia. Median (IQR) ages of patients with these complications were 62 (52–71) for HHS, 36 (21–51) for DKA, 55 (44–66) for hyperglycemia and 52 (37–68) for hypoglycemia, p< 0.0001. The median (IQR) duration of hospital admission for glycemic complications was 5 (3–9) days. Gender distribution of the various glycemic complications were HHS, male: female ratio 1.0: 1.3, DKA, male: female ratio 1.0:1.3, for uncomplicated hyperglycemia, male: female ratio 1.0:1.6, hypoglycemia, male to female ratio, 1.0:1.8. The trends in the proportion of patients with glycemic complications presenting with HHS, DKA, hypoglycemia and hyperglycemia did not increase over the decades from the 1980s to 2010s.

#### End-organ or cardiovascular co-morbidities

Two thousand three hundred and ninety-two (21.0%) diabetic admissions were due to end organ complications. Of these, 503 (18.7%) had peripheral vascular diseases, 377(14.0%) had coronary artery diseases, peripheral neuropathic ulcers (26.4%), 529 nephropathies (18.3%), 282 (10.5%) cerebrovascular diseases. Again 1207(44.8%) had nephropathy and 325(12.0) had peripheral neuropathic ulcers. Overall, the median (IQR) age for patients presenting with end organ complication was 56 (44–67); 60 (52–70) for nephropathy, 55 (45–69) for peripheral vascular disease, 49 (45–58) for coronary artery disease, 61 (44–65) for peripheral neuropathies and 59 (33–66) for cerebrovascular diseases (p <0.001). The median (IQR) for duration of admission was 8 (5–16) compared with 6 (3–11) for those with and without end-organ complications respectively. The median duration of admission for those with nephropathy, peripheral neuropathies, coronary artery diseases, peripheral vascular disease and cerebrovascular disease were, 12 (9–21), 14(8–19), 6 (5–20), 9 (4–19), 11 (7–15) (p< 0.005), respectively. The trends in the numbers for the various end- organ complications did not change significantly over the decades.

### Duration of hospitalisation for diabetes admissions

The median (IQR) duration of hospitalisation for diabetes patients over the study period was 6 days (3–10) days with that for diabetes survivors of 6 days (3–11) compared with 3 days (1–7), p<0.0001 for those who succumbed. The median (IQR) duration of hospitalisation of diabetes patients significantly decreased from 9 (5–15) days in the 1980’s to 8 (4–14) days in the 1990’s, to 6 (3–9) days in the 2000’s and 4 (2–10) days in the 2010s, p<0.0001.

### Diabetes Case Fatality and Its predictors

**Fig 2:**
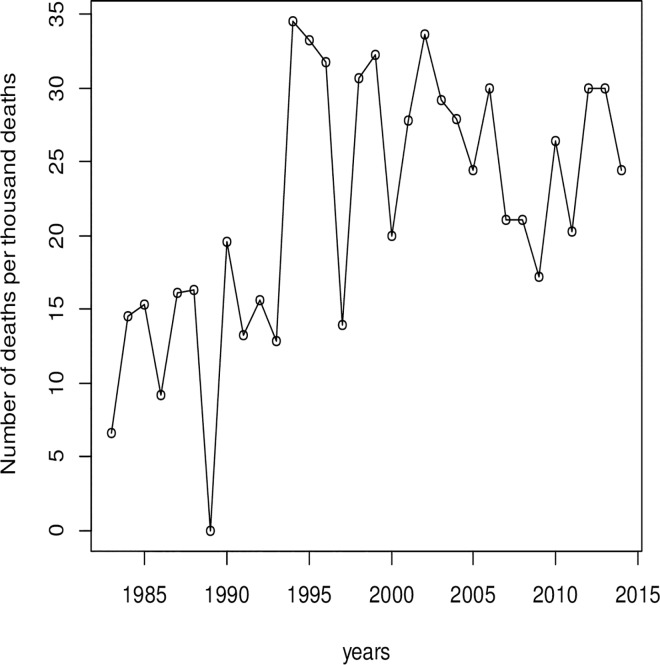
Yearly trend in diabetes deaths per thousand in-patient deaths in central Ghana, 1983–2014.

**Fig 3:**
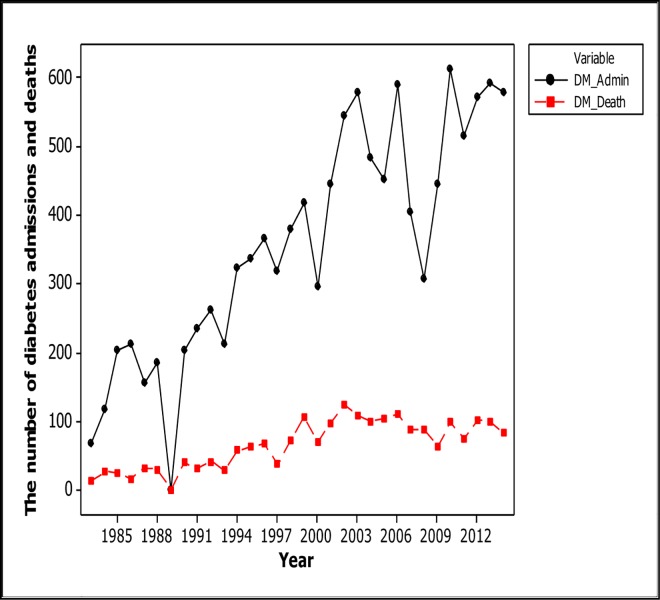
Trends in absolute diabetes admissions and mortality in central Ghana, 1983–2014.

The number of diabetes deaths per 1000 hospital deaths in 1983 was 6.6% and this increased progressively to 30.01 in 2012 as shown in Fig 2.The absolute number of deaths from diabetes increased over the period of analysis as shown in Fig 3 and Table 3. The case fatality rate of diabetes over the study period varied between 7.58 and 28.57 of the diabetes-related deaths, the overall crude 28-day case fatality rate was 18.5%. The trends in diabetes case fatality was however not significant statistically.

**Table 3:** Mortality rates of diabetes at Komfo Anokye Teaching Hospital between 1982 and 2014.

Year	No. of in-patient deaths per year	No. of diabetes deaths per year	Diabetes deaths per 1,000 deaths	Diabetes case fatality
1983	1972	13	6.6	19.12
1984	1797	26	14.5	22.41
1985	1636	25	15.28	12.25
1986	1738	16	9.21	7.58
1987	1992	32	16.06	15.17
1988	2097	30	16.3	14.23
1989	2012		-	-
1990	2161	40	19.61	15.09
1991	2030	31	13.25	12.71
1992	2052	41	15.64	12.5
1993	2182	28	12.83	13.98
1994	1681	58	34.5	17.96
1995	2170	62	33.18	21.36
1996	2145	68	31.7	23.28
1997	2652	37	13.95	20.4
1998	2350	72	30.64	22.68
1999	2485	105	32.25	20.91
2000	3456	69	19.97	24.02
2001	3451	96	27.81	23.28
2002	3693	124	33.58	26.05
2003	3700	108	29.19	18.52
2004	3587	100	27.88	21.28
2005	4260	104	24.41	20.16
2006	3706	111	29.95	22.89
2007	4136	87	21.03	16.89
2008	4174	88	21.08	28.57
2009	3607	62	17.19	13.84
2010	3782	100	26.44	16.34
2011	3649	74	20.28	14.68
2012	3399	102	30.01	19.86
2013	3305	98	29.96	16.53
2014	3442	84	24.4	14.50

As shown in Table 4, the probability of in-patient mortality from diabetes mellitus increased significantly with increasing age, male gender, decade of admission, presence of glycemic complications and end-organ complications at presentation. In the multivariable logistic regression model, significant predictors of mortality were age, aOR of 1.24 (95%CI: 1.16–1.32) among patients >80 years compared to those aged < 20 years, male gender, aOR of 1.45 (95% C.I 1.19–1.61) compared with females, patients admitted in the 2000s compared with those admitted in the 1980s, aOR of 2.07 (95%CI: 1.62–2.63). Furthermore, patients admitted with DKA, HHS and symptomatic hypoglycemia had significantly higher risk of death compared to those with no glycemic complication with aORs of 2.55 (95%CI: 2.12–3.05), 1.52 (95%CI: 1.33–1.73) and 1.64 (95%CI: 1.24–2.17) respectively. So were patients admitted with peripheral vascular disease, coronary artery disease, cerebrovascular disease, nephropathy and peripheral neuropathic ulcers compared with those without complications with aORs of 1.51(95% C. I 1.12–1.21), 3,21(95% C.I 1.91–5.15), 1.52(95% C.I 1.32–1.98), 1.11(95% C.I 1.03–1.23) and 1.31(95% C.I 1.12–1.56) respectively.

**Table 4:** Multiple Logistic regression analysis of predictors of in patient’s mortality for diabetes.

	Unadjusted OR		Adjusted 0R	
	HR (95% CI)	P-value	HR (95%CI)	P-value
**Age**				
each 20 year increase	1.24 (1.16–1.32)	<0.0000	1.23 (1.15–1.32)	<0.0000
**Gender**				
Female	1		1	
Male	1.65 (1.49–1.83)	<0.0000	1.45 (1.19–1.61)	<0.0000
				
**Decade**				
1980's	1		1	
1990's	1.25 (0.96–1.62)	0.1	1.16 (0.89–1.52)	0.26
2000's	2.07 (1.62–2.66)	<0.0000	1.56 (1.21–2.01)	0.0006
2010's	1.54 (1.18–2.01)	0.002	1.20 (0.89–1.62)	0.23
				
**Glycemic complication**				
No Glycemic complication	1		1	
HHS	1.67 (1.47–1.90)	<0.0000	1.52 (1.33–1.73)	<0.00001
DKA	2.55 (2.12–3.05)	<0.0000	2.67 (2.21–3.21)	<0.00001
Hypoglycemia	1.65 (1.25–2.19)	0.0004	1.64 (1.24–2.17)	0.0006
**End organ complication**				
Peripheral neuropathy with Foot ulcers	1.42 (1.20–1.65)	<0.0000	1.31 (1.12–1.56)	<0.00001
Nephropathy	1.20 (1.11–1.29)	<0.0000	1.11 (1.03–1.23)	0.0003
Cerebrovascular disease	1.78 (1.42–2.22)	<0.0000	1.52 (1.32–1.98)	<0.00001
Coronary artery disease	4.00 (2.91–5.52)	<0.0000	3.21 (1.91–5.15)	<0.00001
Peripheral vascular disease	1.43 (1.28–1.60)	<0.0000	1.15 (1.12–1.21)	0.0006

## Discussion

To the best of our knowledge, this is the first study to examine the longitudinal trajectory of hospital admission and mortality rates for diabetes in the middle belt of Ghana. We have shown that diabetes admissions have increased significantly in the central belt of Ghana over the last three decades. This is reflected by both an increase in the absolute numbers of diabetes admissions and the rate of diabetes admissions expressed as the number of diabetes admissions per total admissions in the hospital over the period. To calculate rates of diabetes admissions, we chose the denominator of total hospital admissions (not total adult medical admissions) to gain a global perspective on the increasing burden of diabetes admissions which is an emerging challenge in most hospitals across Africa. In a previous hospital-based study in the southern belt of Ghana, the percentage of medical admissions due to diabetes increased almost two fold from 3.5% in the mid-1970s to 6.4% in the 1990s representing an increase from 2% to 12% of adult medical admissions accounted for by diabetes [13,14]. Similarly, in Ethiopia, diabetes admissions increased from 51 per 100,000 in 2005 to 245 per 100 000 in 2009 [17]. Our longitudinal appraisal in the middle belt of Ghana shows an increment of nearly 633% in the rate of diabetes admissions between 1983 and 2014. This is consistent with the increasing prevalence of diabetes in Low and Middle Income Countries (LMIC) including countries in sub-Saharan Africa [1,2,17,18]. The rising rates of diabetes admissions and increasing mean age of admitted patients is reflective of the increasing longevity of the citizenry of Ghanaians attended by modest improvements in the socio-economic status leading to urbanisation and adoption of western life-styles that are serving as undercurrents for the epidemiological transition being experienced by many LMICs [19,20]. Accordingly, the population prevalence of vascular risk factors in particular obesity, physical inactivity, systemic arterial hypertension has escalated in several countries across Africa culminating in the rising burden of diabetes and other non-communicable diseases [20–27]. For instance, about four in five adult Ghanaians has been recorded not to engage in enough physical activity [20–22]. On the other hand, obesity rates have increased significantly over a relatively short period of time [20,23,24]. From a recorded prevalence of 25.5% in 2003, obesity rates have increased to 30.5% of the general adult population by the year 2008 [25]. Additionally, unhealthy eating patterns are becoming common among Ghanaian communities. In the World Health Survey 2002–2003, fruits and vegetable consumption in Ghana were the lowest among 52 mainly low- and middle-income countries including 19 African nations [24,25]. This have reflected in increases in the prevalence of hyperlipidemia among Ghanaians with current estimates ranging between 17 and 23 percent [26,27]. In most developing countries, the burden of hypertension is on the ascendency [28–30]. In Ghana, estimates currently put the prevalence of hypertension as high at 36% [25]. To add to this, recent estimate indicates a smoking prevalence around 10% among Ghanaians, with higher rates seen in men than women [25]. The current rate of smoking in men is high compared to rates on the continent [31]. The increasing prevalence of these risk cardiovascular risk factors largely explains the increasing prevalence of diabetes in sub-Saharan Africa and the increasing trends recorded in this study. Overall female gender dominated the number of admitted cases of diabetes in this cohort. This is largely explained by the higher prevalence of obesity and physical inactivity among women [21–24]. Additionally, women live relatively longer than men and as a result are exposed to diabetes and other non-communicable diseases [15]. An increasing trend in the mean age of diabetes patients at hospitalisation was evident over the period of the study; from a mean ± SD age of 48.9 ± 17.7 in 1980s to 58.5 ±16.9 in 2010s. Female diabetes patients tended to be older than male patients with the difference in mean age attaining statistical significance in 3 out of the 31 years under review. The rising rates of diabetes admissions and increasing mean age of admitted patients is reflective of the increasing longevity of the citizenry of Ghanaians most especially females [23,29,30].

The 28-day in-patient mortality from diabetes admissions of 18.5% observed in this study ranks among the highest in the published literature. In hospital-based studies, a 28-day diabetes case fatality of 11.8% in Korea and 17% Taiwan was recorded [32,33]. In the United Kingdom and New Zealand both industrialised countries, in patient mortality rates of 3.9% and 2.4% have been recorded [34,35]. Given that hospital admission for acute diabetic complications is often avoidable and mortality associated with it represents sentinel health events of inadequate outpatient care, outpatients care in terms of diabetes patient education, reduction in modifiable risk factors associated with diabetes and appropriate glycemic control is not optimum in Ghana based on results from this study. The high rate of in-patient mortality predicted by end-organ complications in this study can also indicate either delayed presentation for orthodox management in a population where awareness of symptoms and signs of diabetes is limited with patients often resorting to alternative treatments initially for a true medical emergency which diabetes is or could represent a scenario where patients with severe diabetes gets referred to a tertiary institution for care late.

A multivariable analysis conducted in the current study revealed diabetes fatality was highest among patients aged 80 years and above, males and was worst among patients admitted in the 2000–2009 period compared with 1983–1989. In the aged, other co- morbidities usually exist and as such are more likely to die from diabetes complications as shown in this study. Even though diabetes admissions have increased over the past 30 years, hospital facilities have not undergone a corresponding expansion and duration of hospital stay for diabetes patients have shortened significantly resulting in the increasing numbers of diabetes admissions. Multivariate analysis also revealed that diabetes mortality was more likely in patients presenting with glycemic complications such as ketoacidosis, hyperosmolar state and hypoglycemia as well as end-organ complications. This observation captures the lack of resources and trained personnel as well as absence of protocols for management of diabetic emergencies and end–organ complications over the past three decades.

Data on blood glucose, lipids, blood gas analysis were missing from available records and accuracy of diabetes could not be adequately determined. This study is also limited by the lack of detailed demographic and other clinical variables which could explain the increasing incidence of diabetes admissions as well as in-patient mortality. Lastly, the specific causes of diabetes-related deaths were also not ascertained. These limitations raise important questions for further studies in helping to elucidate the undercurrents driving the diabetes epidemic in Ghana and the poor clinical outcomes in central Ghana.These notwithstanding, we have captured and presented the devastating short-term outcomes of diabetes admissions which calls for setting up of dedicated diabetes units where diabetes management protocols suitable and appropriate for countries with limited resources could be implemented. Again aggressive patient education should be instituted together with aggressive reduction of risk factors for diabetes. Improving diabetes clinic availability to patients of different groups and socioeconomic backgrounds as well as increasing their involvement in education programs will reduce the risk of acute glycemic and end-organ complications of diabetes. Efforts at further expansion of the diabetes units, training of health care personnel and formation of diabetes care teams would be crucial in mitigating the devastating in-patient outcomes whilst vigorous public education measures at awareness of diabetes risk factors, symptoms and signs for prompt attention need to be pursued with ardent vigour.

In conclusion, we have shown in this study that the absolute and proportionate numbers of diabetes admissions and mortality have increased steadily over the past three decades in central Ghana. There is a progressive increase in the mean age of diabetes patients over the time period of the study with one in a fifth of diabetes patients succumbing after admission. These observations should prompt urgent concerted and coordinated efforts at arresting the burgeoning diabetes epidemic in sub-Saharan Africa.

## Supporting Information

S1 Data Set Diabetes admissions and mortality in Central Ghana. (XLSX)
